# A Novel UDP-Glycosyltransferase of* Rhodiola crenulata* Converts Tyrosol to Specifically Produce Icariside D2

**DOI:** 10.1155/2018/7970590

**Published:** 2018-06-20

**Authors:** Zhihua Liao, Fei Qiu, Junlan Zeng, Li Gu, Bangjun Wang, Xiaozhong Lan, Min Chen

**Affiliations:** ^1^TAAHC-SWU Medicinal Plant Joint R&D Centre, Xizang Agricultural and Husbandry College, Nyingchi of Tibet 860000, China; ^2^Key Laboratory of Eco-Environments in Three Gorges Reservoir Region (Ministry of Education), Chongqing Key Laboratory of Plant Ecology and Resources Research in Three Gorges Reservoir Region, SWU-TAAHC Medicinal Plant Joint R&D Centre, School of Life Sciences, Southwest University, Chongqing 400715, China; ^3^College of Pharmaceutical Sciences, Key Laboratory of Luminescent and Real-Time Analytical Chemistry (Ministry of Education), Southwest University, Chongqing 400715, China

## Abstract

*Rhodiola crenulata* is a Tibetan native herbal plant belonging to the family of Crassulaceae, which produces the pharmaceutical icariside D2 with the activities of inhibiting angiotensin-converting enzyme and killing leukemia cancer cells. In this study, we functionally characterized a novel UDP-glycosyltransferase (RcUGT1) that converted tyrosol to specifically produce icariside D2 from* R*.* crenulata* at molecular and biochemical levels.* RcUGT1* was highly expressed in flowers and roots, while the icariside D2 content was much higher in stems than that in other organs, suggesting the potential translocation of icariside D2 from flowers and roots to stems. The high production of icariside D2 in stems provided a reasonable suggestion to farmers to harvest stems instead of roots for icariside D2 production. Enzymatic assays of recombinant RcUGT1 indicated that it converted tyrosol to specifically form icariside D2, with the values of* K*m 0.97±0.10 mM,* V*max 286±8.26 pKat/mg,* K*cat 0.01552 s^−1^, and* K*cat/*K*m 159.55 s^−1^ M^−1^. Functional identification of RcUGT1 facilitated the icariside D2 production through metabolic engineering in plants or synthetic biology in microbes.

## 1. Introduction

**Figure 1:**
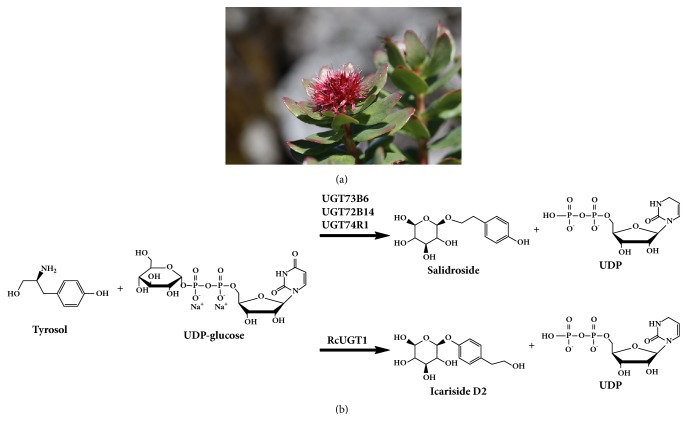
*Rhodiola crenulata* and the biosynthesis of salidroside and its isomer icariside D2. (a) The flowering plant of* Rhodiola crenulata*. (b) UDP-glycosyltransferases (UGTs) convert tyrosol to salidroside and icariside D2.

*Rhodiola crenulata*, a Tibetan native herbal plant belonging to the family of Crassulaceae (Figure 1(a)), has been used as herb and health food to relieve altitude sickness more than 1000 years before by the native people in Tibet Plateau [1]. This magic plant species grows very slowly due to the harsh environments in the high mountains more than 5000 meters, where the ultraviolet radiation is strong, the oxygen concentration is low, the water is precious, and the soil is poor [2]. The modern pharmaceutical sciences reveal that the polyphenol glucosides such as salidroside in* R*.* crenulata* are the biologically active compounds for relieving altitude sickness, reinforcing immunity, antifatigue, and so forth [3]. Recently, it was found that icariside D2 (the isomer of salidroside) was helpful to inhibit the activity of angiotensin-converting enzyme [4] and potent to kill leukemia cancer cells HL-60 [5]. Traditionally, the perennial roots of* R*.* crenulata* are harvested to produce these pharmaceutical compounds [6]. The wild plants of* R*.* crenulata* are the most important source for producing these chemicals, and unfortunately the artificial cultivation of* R*.* crenulata* is not available, while the demand for* R*.* crenulata* has been growing. Consequently,* R*.* crenulata* is endangered.

It is important to find alternative methods to produce salidroside and icariside D2. Metabolic engineering, as well as synthetic biology, might be a promising way to do this, which is dependent on elucidation of their biosynthetic pathway. Tyrosine decarboxylase (TYDC) is the first rate-limiting enzyme involved in tyrosol glycoside biosynthesis. TYDC catalyzes tyrosine decarboxylation to form tyramine as a key intermediate for tyrosol glycosides [6]. Overexpression of tyrosine decarboxylase enhanced salidroside and tyrosol accumulation in* Rhodiola* hairy root cultures [7]. Three specific UDP-glycosyltransferases (UGTs) were reported to convert tyrosol into salidroside (Figure 1(b)) in* Rhodiola sachalinensis* [8], and overexpression of one of them (RsUGT73B6) promoted salidroside production [9]. RsUGT73B6 was further used to produce salidroside in engineering* E*.* coli* through the strategy of synthetic biology [10]. However, the enzyme required for converting tyrosol into icariside D2 in* R*.* crenulata* has not been identified. In this study, we characterized a novel UDP-glycosyltransferase (RcUGT1) that converted tyrosol to specifically produce icariside D2 from* R*.* crenulata* at molecular and biochemical levels.

## 2. Experimental Procedures

### 2.1. Plant Materials and Chemicals

Because* Rhodiola crenulata* is an endangered plant species, the official approval is necessary to collect the plant materials. The whole plants of* Rhodiola crenulata *were collected from the northern area of Tibet Himalaya Mountain in July of 2011 [6], under the official approval of Tibet Sciences and Technology Committee (Lhasa, Tibet, China). The different organs, including flowers, leaves, stems, and roots, were immediately immersed in liquid nitrogen for future use [6]. The authentic icariside D2 was purchased from BioBioPha (Yunnan, China). UDP-glucose and salidroside were purchased from Sigma-Aldrich (Louisiana, USA). Tyrosol was purchased from Aladdin (Shanghai, China).

### 2.2. Gene Cloning and Expression Analysis

Total RNAs were isolated from the different organs of* R*.* crenulata* using the RNAsimple Kit according to the manufacturer's protocol (Tiangen Biotech, Beijing, China). The cDNAs were synthesized using the FastKing RT Kit (Tiangen Biotech, Beijing, China). The conserved core sequence of* RcUGT1* was amplified using a pair of primers, dF and dR. Based on the sequence core sequence of* RcUGT1*, the cDNA ends were cloned using RACE technique. The 3′ RACE and 5′ RACE of* RcUGT1* were, respectively, performed according to the manufacturer's protocol (ClonTech, CA, USA). Each PCR product was subcloned into the pMD-18T vector and sequenced. The corresponding fragments of* RcUGT1* were assembled to generate the putative full-length cDNA and the coding sequence was amplified using a pair of gene-specific primers, pF and pR. Real-time quantitative PCR was used to analyze the tissue profile of RcUGT1 on an iQ5 system (Bio-Rad, USA), as described before [6]. The primers used in this study were listed in Table 1.

### 2.3. Bioinformatic Analysis

The sequences analysis was online performed at the NCBI websites. The multiple alignments of RcUGT1 and other UGTs were aligned using the Vector NTI software. The phylogenetic tree was constructed by MEGA 5 [11] using the Neighbor-joining methods [12] with 10000 repeats.

### 2.4. Detection of Icariside D2 in Plant Tissues

The contents of icariside D2 were analyzed according to the previously reported methods [12]. The plant materials were completely dried at 40°C and then finely powdered. The powdered plant materials were dipped in distilled water for 15 min and then autoclaved at 130°C for 30 min. The autoclaved materials were shaken at 37°C for 1h at the speed of 200 rpm. The supernatants were collected after centrifuge (12000 rpm) and then filtered through 0.22 *μ*M film, which could be used for analysis of icariside D2 using high performance liquid chromatography (HPLC).

### 2.5. Purification of the His-Tagged RcUGT1 from Engineered E. coli

The coding sequence of* RcUGT1* was inserted into prokaryotic expression vector pQE30 using the restriction enzymes* BamH*I and* Sac*I to generate the recombinant vector of pQE30-RcUGT1. The recombinant pQE30-RcUGT1 vector was introduced into* E*.* coli* M15 for expression. After induction by 0.5 mM isopropyl *β*-D-1-thiogalactopyranoside (IPTG) for 4 h at 25°C, then, the cells were harvested by centrifugation at 8000 rpm for 5 min. About 5 g cell pellets were resuspended in 50 ml of Buffer A (50 mM sodium phosphate, pH 8.0, 300 mM NaCl, and 5 mM imidazole) with lysozyme (1 mg/mL). After standing for 30 min on ice, this cell suspension was lysed by sonication. The lysate was obtained by centrifugation at 12000 rpm for 15 minutes. The recombinant protein could be detected on SDS-PAGE gel with amounts enough for enzymatic assay in the supernatants. Then Ni^2+^-NTA resin was used to bound His-tagged RcUGT1. Next, the resin eluted impurity with Buffer B (20 mM imidazole, 300 mM NaCl, 50mM NaH_2_PO_4_, and pH 8.0) about 50 ml. Then the His-tagged RcUGT1 was eluted with wash buffer (80 mM imidazole, 300 mM NaCl, 50mM NaH_2_PO_4_, and pH 8.0). Fractions were analyzed by SDS-PAGE.

### 2.6. Enzyme Assays of RcUGT1

The purified RcUGT1 protein was used for enzymatic assay in the 500 *μ*L reaction buffers containing 50 mM Tris-HCl, 2 mM tyrosol, and 2 mM UDP-glucose [8]. 20 *μ*g of RcUGT1 was used to investigate the kinetics parameters at pH 8.6. The reaction was performed for 30 min at 30°C and then stopped using 500 *μ*l methanol in an ice box [8]. After centrifugation at 15000 g for 10 min at 4°C, the reaction products were analyzed by HPLC as the reported methods [8]. The separation of samples was detected by UV absorbance at 225 nm. The mobile phase contained solvent A (water with 20 mM ammonium acetate and 0.1% formic acid) and solvent B (methanol). The flow rate was 1 ml/min. The boiled RcUGT1 was used as control in enzymatic assay. The mass spectrum of icariside D2 was analyzed on the system of LC-MS 8030 (SHIMADZU, Japan) using the Grace Alltech Altima C18 5 *μ*M column.

## 3. Results and Discussion

### 3.1. Molecular Cloning and Sequence Analysis

**Figure 2:**
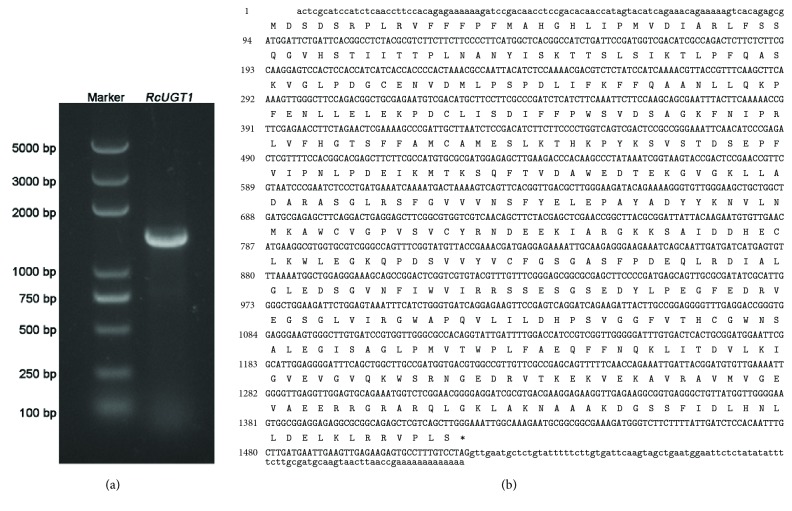
Molecular cloning of the full-length cDNA of RcUGT1. (a) RcUGT1 in the agarose gel. (b) The cDNA sequence of RcUGT1, of which coding sequence was shown in capital bold letters and the untranslated regions in normal letters. The stop codon was marked with an asterisk.

**Figure 3:**
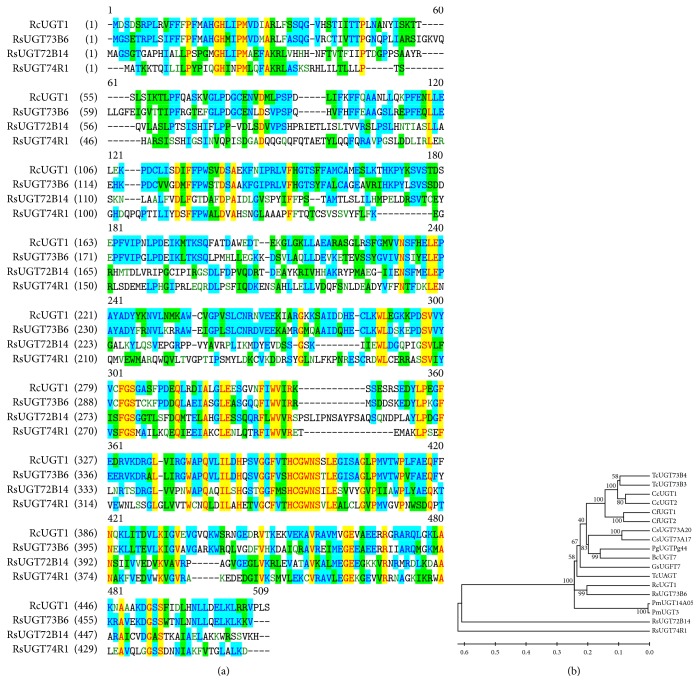
The multiple alignments and phylogenetic analysis of plant UGTs. (a) The multiple alignments of RcUGT1 and the three UGTs of R. sachalinensis. The PSPG-box (plant secondary product glycosyltransferase box) was boxed. (b) The phylogenetic analysis of plant UGTs. The numbers represented the bootstrap values given by 1000 repeats. The scale represented the genetic distance.

The core fragment of* RcUGT1* was 790 bp in length and the BLASTN result suggested that it belonged to the UGT family. Based on the 790-bp core fragment, the 708-bp 3′ end, as well as the 718-bp 5′ end of* RcUGT1*, was amplified using RACE technique, respectively. The full-length cDNA of RcUGT1 was generated through assembling the 3′ end, the core fragment, and 5′ end and its physical sequence was further isolated (Figure 2(a)).* RcUGT1* had a 1425-bp coding sequence encoding a polypeptide of 474 amino acids with the calculated molecular weight of 53 kDa and isoelectric point of 6.12 (Figure 2(b)). The sequence of* RcUGT1* was deposited in GenBank (Accession Number: MH299424). The sequence analysis showed that RcUGT1 belonged to the glycosyltransferase B-type (GT-B) super family. At the amino acid level, RcUGT1 exhibited 24.7% to 62.5% identity to the three reported UGT proteins (Figure 3(a)), including RsUGT72B14, RcUGT74R1, and RsUGT73B6 of* R*.* sachalinensis* [8]. All the three UGTs of* R*.* sachalinensis* showed the enzymatic activity of conversion of tyrosol to salidroside [8]. According to the results given by sequence comparison (Figure 3(a)) and the phylogenetic tree (Figure 3(b)), RcUGT1 showed relatively close relationship to RsUGT73B6. The 44-amino-acid C-terminal signature motif, named PSPG-box (plant secondary product glycosyltransferase box), was found in RcUGT1, as well as in the three reported* R*.* sachalinensis *UGT proteins (Figure 3(a)). In the PSPG-box, RcUGT1 contained two highly conserved motifs WAPQ and HCGWNS (Figure 3(a)) present in 95% of UGT sequences [13], which were essential for the enzymatic activities of glycosyltransferases due to their crucial roles in the recognition and binding of different aglycones and in the sugar donor specificity [14]. These bioinformatics analysis results suggested that RcUGT1 might catalyze the glycosylation of tyrosol to given corresponding product.

### 3.2. Tissue Profile of RcUGT1

**Figure 4:**
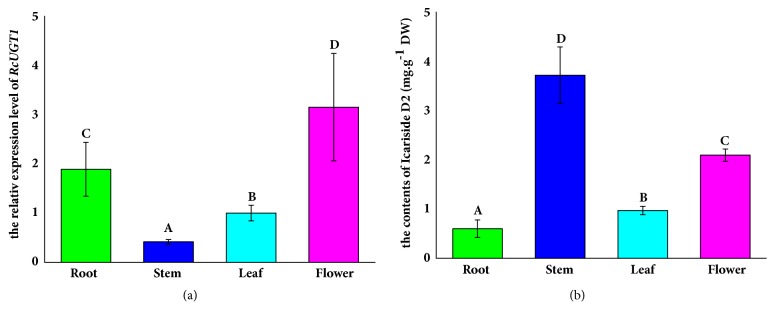
Tissue profile of* RcUGT1* and distribution of icariside D2 in* R. crenulata* organs. The bars on columns represented the standard deviations (n=3). The different letters on column indicated significant difference (p<0.05) given by t-test. (a) The tissue profile of RcUGT1. (b) The icariside D2 contents in different organs.

Generally, the biosynthesis genes are spatially and temporally expressed in plants to regulate the biosynthesis of target metabolites. In* R*.* crenulata*,* RcTYDC*, the first committed enzyme gene, was highly expressed in stems [6]. The tissue profile of* RcUGT1* was analyzed using real-time quantitative PCR. The results demonstrated that* RcUGT1* was expressed in flowers, leaves, stems, and roots but at different levels (Figure 4(a)). The* RcUGT1* transcript level was high in flowers and roots, moderate in leaves, and low in stems.* RcUGT1* expression level in flowers, leaves, and roots was, respectively, 7.6-, 2.4-, and 4.5-fold higher than that in stems. These data indicated that RcUGT1 mainly worked in flowers and roots of* R*.* crenulata*. According to the previous report, the salidroside-producing UGT gene of* R*.* crenulata* was highly expressed in stems, suggesting that it preferably worked in stems [6]. The different tissue profiles between* RcUGT1* and the salidroside-producing UGT gene implied their potentially functional difference from each other.

### 3.3. Distribution of Icariside D2 in R. crenulata Organs

The distribution pattern of icariside D2 was also investigated using HPLC in flowers, leaves, stems, and roots of* R*.* crenulata*. The HPLC analysis indicated that the four types of organs contained icariside D2 at different levels. Flowers, leaves, stems, and roots produced icariside D2 at the levels of 2.09 mg/g dry weight (DW), 0.97 mg/g DW, 3.71 mg/g DW, and 0.59 mg/g DW, respectively. The highest content of icariside D2 was found in stems, where* RcUGT1* was expressed at the lowest level among the four types of organs (Figure 4(b)). The discrepancy between* RcUGT1* expression and icariside D2 content in stems strongly suggested that the translocation of icariside D2 might exist in this plant and that there might be other uncharacterized UGTs converting tyrosol into icariside D2 in* R*.* crenulata*. In fact, the metabolite translocation is often found in diverse plants. For example, the tropane alkaloids including hyoscyamine and scopolamine are specially synthesized in secondary roots and consequently are translocated into airborne parts including fruits, leaves, and stems [15]. Traditionally, the roots of* Rhodiola* are harvested for salidroside production due to high-yield salidroside in roots [8] and obviously this harvest way is not sustainable. Since the stems of* R*.* crenulata* contain icariside D2 at a relatively high level (Figure 4(b)), it is reasonable to suggest the farmers to harvest stems alone for icariside D2 production.

### 3.4. Enzymatic Assays of RcUGT1

**Figure 5:**
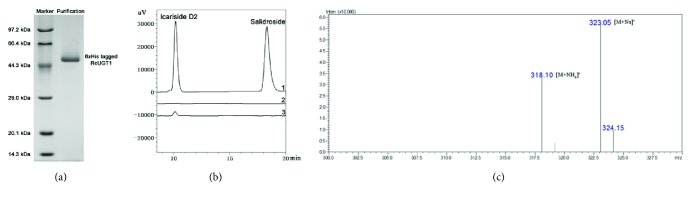
Purification and enzymatic assay of the recombinant His-tagged RcUGT1. (a) Purification of the recombinant His-tagged RcUGT1 from* E. coli*. (b) The HPLC traces of RcUGT1-mediated reactions: 1 represented the authentic icariside D2 and salidroside; 2 was the reaction system of control in which RcUGT1 was denatured by boiling; 3 represented the product (icariside 2) given by RcUGT1. (c) The mass spectrum of icariside D2 produced by RcUGT1.

In order to study whether RcUGT1 converted tyrosol to give its derived glucosides such as icariside D2 and salidroside, we expressed RcUGT1 in* E*.* coli* and purified the recombinant His-tagged RcUGT1 protein from engineering* E*.* coli*. When* E*.* coli* M15 harboring pQE30-RcUGT1 was induced by 0.5 mM IPTG for 4 h at 25°C, the recombinant protein could be detected on SDS-PAGE gel with amounts enough for enzymatic assay in the supernatants. On the SDS-page gel, 6×His-RcUGT1 showed the molecular weight of about 53 kDa (Figure 5(a)), consistent with its calculated molecular weight. RcUGT1 showed very similar molecular weight to the three reported UGT proteins of* R*.* sachalinensis* [8]. Subsequently, the purified RcUGT1 protein was purified through Ni^2+^-NTA resin and then used for enzymatic assay. A single product was detected with the retention time of 10 min in the reaction buffers, consistent with the retention time of the authentic sample of icariside D2 (Figure 5(b)). At the same time, no salidroside was detected. In the control (RcUGT1 was boiled), this specific product was not detected. Then, this specific product was identified using LC-MS, of which the characteristic fragments at the values of* m*/*z* 318 and* m*/*z* 323 (Figure 5(c)) were consistent with the reported* m*/*z* values of icariside D2 [16]. When these results given by HPLC and MS were considered together, it was concluded that RcUGT1 converted tyrosol to specifically form icariside D2, which was different from the three reported* R*.* sachalinensis* UGTs that converted tyrosol to salidroside.

### 3.5. Enzymatic Kinetics of RcUGT1

**Figure 6:**
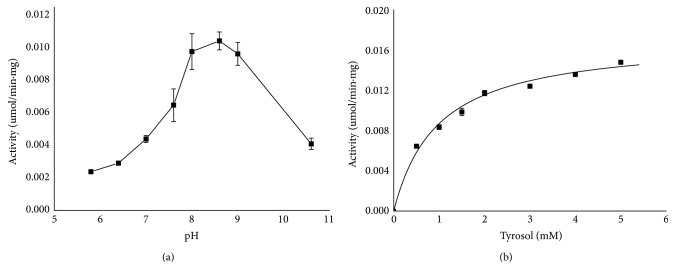
The kinetics analysis of RcUGT1. (a) The optimum pH for RcUGT1. (b) The kinetics analysis of RcUGT1 at optimal pH.

The previous publication studied the enzymatic activities of the three UGTs of* R*.* sachalinensis* at pH 7.5 alone, without optimizing the pH conditions [8]. To determine the optimal pH for RcUGT1, the enzymatic activity was investigated, respectively, at pH from 5.8 to 10.6. RcUGT1 had the maximum activity at pH 8.6 (Figure 6(a)). For example, the activity of RcUGT1 was 4.37-fold higher at pH 8.6 than that at pH 5.8, respectively. Finally, the kinetic parameters of RcUGT1 were investigated in the same reaction buffers described above by changing the concentration of tyrosol from 0.5 mM to 5 mM at the optimal pH 8.6 (Figure 6(b)). The* K*m value of RcUGT1 for tyrosol was 0.97 ± 0.10 mM (Table 2). According to the previous reports, the* K*m values of the three reported UGTs of* Rhodiola sachalinensis*, catalyzing tyrosol to form salidroside, were, respectively, 4.7 *μ*M, 172.4 *μ*M, and 54.3 *μ*M [6]. By comparison of the* K*m values of these enzymes, RcUGT1 exhibited lower affinity for the substrate tyrosol than the reported three enzymes. The* V*max value of RcUGT1 for tyrosol was 286±8.26 pKat/mg, much higher than that of RsUGT72B14 (57.8 pKat/mg), and slightly higher than that of RsUGT73B6 (249.8 pKat/mg), but at the very similar level of RcUGT74R1 (293.1 pKat/mg). The* V*max analysis suggested that the velocity of RcUGT1 was higher than those of RsUGT72B14 and RsUGT73B6, but similar to that of RcUGT74R1. Although the* K*cat values of the three other UGTs were not reported, we detected the* K*cat of RcUGT1. The* K*cat value of RcUGT1 was 0.01552 s^−1^. The* K*cat/*K*m value of RcUGT1, representing the catalytic efficiency, was 159.55 s^−1^ M^−1^. The enzymatic assays suggested that RcUGT1 might be a good candidate to specifically produce icariside D2 through metabolic engineering or synthetic biology.

## 4. Conclusion

In summary, we have characterized a novel enzyme that catalyzes the glycosylation of tyrosol to specifically form icariside D2, which is potentially useful in the production of icariside D2 through metabolic engineering and synthetic biology. The stems of* R*.* crenulata* produce icariside D2 at higher level than other organs, providing a reasonable suggestion to farmers to harvest stems instead of roots for icariside D2 production.

## Figures and Tables

**Table 1:** The primers used in this study.

**Primer**	**Sequence(**5′-3′**)**	**Aim**
dF	GGCATCCCGCGGCTNGTNTTYCAYG	Cloning of the core sequence
dR	CGGTCACCAGCTTCTCGTTGWARAAYTGNTC	Cloning of the core sequence
Primer 3′-1	GCCGGACTCGGTCGTGTACGT	3′ RACE
Primer 3′-2	GAGAAGTTCCGAGTCAGGAT	3′ RACE
Primer 5′-1	GACCATTTCTGCACTCCAACCT	5′ RACE
Primer 5′-2	GAACAACGGCCACDTCACCAT	5′ RACE
pF	CGCGGATCCATGGATTCTGATTCACGGCCT	Vector construction
pR	CGCGGTACCCTAGGACAAAGGCACTCTTCT	Vector construction
Primer qF	ACTCATTGCGGATGGAAT	qPCR
Primer qR	CCTTCTCAACCTTCTCCTT	qPCR

**Table 2:** The kinetics parameters of UGT enzymes.

**Enzyme**	**Substrate**	**Product**	***K*m **	***V*max **	***K*cat **	***K*cat/*K*m **	**References**
			**(** ***μ*** **M)**	**(pKat mg** ^**-1**^ **)**	**(s** ^**-1**^ **)**	**(s** ^**-1**^ ** M** ^**-1**^ **)**	
RcUGT1	Tyrosol	Icariside D2	972.75±104.31	286±8.26	0.01552±0.00044	159.55	this study
RsUGT72B14	Tyrosol	Salidroside	4.7±0.35	57.8±3.2	-	-	Yu et al., 2011
RsUGT74R1	Tyrosol	Salidroside	172±14.1	293.1±14	-	-	Yu et al., 2011
RsUGT73B6	Tyrosol	Salidroside	54.3±4.9	249.8±13	-	-	Yu et al., 2011
